# Evaluation of immunoglobulin purification methods and their impact on quality and yield of antigen-specific antibodies

**DOI:** 10.1186/1475-2875-7-129

**Published:** 2008-07-14

**Authors:** Elke S Bergmann-Leitner, Ryan M Mease, Elizabeth H Duncan, Farhat Khan, John Waitumbi, Evelina Angov

**Affiliations:** 1 US Military Malaria Vaccine Program, Division of Malaria Vaccine Development, WRAIR, Silver Spring, MD, USA; 2 Walter Reed Project, Kenya Medical Research Institute, Kisumu, Kenya

## Abstract

**Background:**

Antibodies are the main effectors against malaria blood-stage parasites. Evaluation of functional activities in immune sera from Phase 2a/b vaccine trials may provide invaluable information in the search for immune correlates of protection. However, the presence of anti-malarial-drugs, improper collection/storage conditions or concomitant immune responses against other pathogens can contribute to non-specific anti-parasite activities when the sera/plasma are tested *in vitro*. Purification of immunoglobulin is a standard approach for reducing such non-specific background activities, but the purification method itself can alter the quality and yield of recovered Ag-specific antibodies.

**Methods:**

To address this concern, various immunoglobulin (Ig) purification methods (protein G Sepharose, protein A/G Sepharose, polyethylene glycol and caprylic acid-ammonium sulphate precipitation) were evaluated for their impact on the quality, quantity and functional activity of purified rabbit and human Igs. The recovered Igs were analysed for yield and purity by SDS-PAGE, for quality by Ag-specific ELISAs (determining changes in titer, avidity and isotype distribution) and for functional activity by *in vitro *parasite growth inhibition assay (GIA).

**Results:**

This comparison demonstrated that overall polyethylene glycol purification of human serum/plasma samples and protein G Sepharose purification of rabbit sera are optimal for recovering functional Ag-specific antibodies.

**Conclusion:**

Consequently, critical consideration of the purification method is required to avoid selecting non-representative populations of recovered Ig, which could influence interpretations of vaccine efficacy, or affect the search for immune correlates of protection.

## Background

Serological analysis of humoral responses is crucial for vaccine evaluation as well as epidemiological studies for identification of immune correlates. For both *in vivo *(when passively transferred) and *in vitro *applications, immunoglobulins need to be purified in order to not only eliminate potential pathogens but also to eliminate non-specific effects of other serum components such as complement, oxidative radicals, cytokines and drugs. Depending on the application, standard purification protocols are either geared towards large scale purifications under GMP-conditions of polyclonal Ab from immune individuals [1-4] or towards the isolation of monoclonal Ab from either biological fluids or culture supernatant for basic research study or passive immunotherapy [5-8].

The majority of methods described in the literature are based either on binding to certain subclasses of immunoglobulins (Igs) by molecules such as Protein A and G [7] or on precipitating proteins in the size range of Igs with reagents such as ammonium sulfate, (NH_4_)_2_SO_4_, [6], caprylic acid/(NH_4_)_2_SO_4 _[9] or polyethylene glycol (PEG) [10,11]. Some methods – here the exception being (NH_4_)_2_SO_4 _and PEG protocols – require an acidic condition between pH 2–4 which can have a negative impact on the quality, integrity and function of the purified antibody molecules. In addition, the efficiencies of recovering a representative pool of antibodies can vary by method.(*e.g*. for human Igs Protein A binds preferentially to IgM and IgG isotypes, but not to the IgG3 subclass). This is of particular interest for disease models in which IgG3 acts as a marker of exposure, such as in protozoan infections [12-14] as well as for the mounting evidence that blood-stage specific IgG3 may be associated with protective immunity against malaria depending on the target antigen [15-17]. In addition, IgG3 has been shown to be the most efficient Ig isotype in mediating antibody-dependent cellular inhibition (ADCI) at least *in vitro *[18]. Similar selectivity for isotypes has been reported for murine as well as rat antibodies. Igs from some species do not purify at all when using Protein A or G [19].

Very limited information is available in the literature on how the integrity and functional activity of antibodies are affected by various purification methods; thus, a formal comparison is needed to evaluate any differences. One major focus point in malaria vaccine development is the analysis of immune responses against the blood stage parasite of *Plasmodium falciparum*. Thus, model systems were established based on sera from rabbits immunized with a recombinant 42 kDa C-terminal portion of Merozoite Surface Protein 1 (MSP-1p42) and plasma/serum from malaria-exposed residents and respective malaria-naïve rabbit- and human control sera. The methods chosen for comparison were: (a) depletion of serum proteins by caprylic acid followed by precipitation of Ig using (NH_4_)_2_SO_4_, designated CA-AS; (b) enrichment of Ig using PEG; (c) specific binding of IgG using Protein G Sepharose; and (4) specific binding of IgM and IgG isotypes using Sepharose coupled with Protein A/G. The recovery from each method was determined by UV spectrophotometry and by Ag-specific ELISAs. The integrity of each Ig preparation was tested by SDS-gel electrophoresis and the ability of the isolated Ig to bind to their specific antigen (Ag) was measured in MSP-1p42 specific ELISAs. Potential changes in the Ag-specific titer, isotype distribution and Ab avidity were also determined by ELISA. Lastly, conservation of biological function of purified Ig after separation with the respective methods was tested by *in vitro *parasite growth inhibition assays (GIA). This led to the observation that the main difference between the methods was in the yield of recovered Igs as reflected in changes in the MSP-1p42-specific Ab titer. Overall, for rabbit Igs, the purity was not significantly different, as judged by SDS-gel electrophoresis, while for humans, it was highest using Protein G, CA-AS and PEG. Biological activity of the recovered Igs was retained for all preparations except for the PEG preparation of the rabbit Igs and less for the Protein G and A/G preparations of the human Igs. Thus, these observations led to the conclusion that considerable qualitative and quantitative differences exist between the Ig purification methods as a function of the species of origin and thus suitable purification methods must be chosen to avoid experimental artifacts which could lead to mis-interpretation of vaccine effects or mis-characterization of immune correlates.

## Materials and methods

### Source of MSP-1p42 specific Ab

New Zealand White rabbits (Spring Valley Laboratories, Poolesville, MD) were subcutaneously immunized four times with recombinant MSP-1p42 of the FVO strain (36 μg per immunization) [20] emulsified in complete (for prime) or incomplete (for three booster immunizations) Freund's adjuvant (Sigma Aldrich, St. Louis, MO). Rabbits were exsanguinated four weeks after the last immunization and the serum aliquots for this study were pooled. Control sera were prepared by pooling sera from two rabbits immunized three times with 50 μg of reduced/alkylated MSP-1p42 (FVO) in complete/incomplete Freund's adjuvant. Human malaria-naïve sera were commercially obtained from Interstate Blood Bank (Memphis, TN). The malaria immune plasma samples were obtained from adults living in a malaria endemic region of western Kenya or from an individual having known immunity against *P. falciparum *living in Gabon provided as a kind gift from Prof. Alain Vernes (Bio Merieux, France) [21].

### Immunoglobulin purification methods

(1) Enrichment of total immunoglobulin by a two step sequential caprylic acid depletion of serum proteins followed by ammonium sulfate precipitation of immunoglobulins was performed as essentially described earlier [6,22].

(2) Selective enrichment of immunoglobulins using the modified volume exclusion action of PEG from the SEP-EASE (Polyclonal) Purification kit (Clinical Science Products, Mansfield, MA) was according to the manufacturer's instructions.

(3) Specific binding of IgM and IgG to Ultralink™ Immobilized Protein A/G Sepharose slurry (Pierce, Rockford, IL). Protein A/G binds to all human IgG subclasses. For this protocol, serum/plasma samples were diluted 1:10 using ImmunoPure^® ^(G) IgG Binding Buffer (Pierce) and batch-bound to 500 μl of pre-equilibrated 50% Ultralink™ Immobilized Protein A/G Sepharose overnight at 4°C. The slurry was applied to a column by gravity flow and allowed to pack. The flow-through was batch bound a second time for 45 min at 22°C (RT) and again applied to a chromatographic column. Bound Ig was eluted, at 500 μl per fraction, from the column using ImmunoPure^® ^(G) IgG Elution Buffer (Pierce) into tubes containing 20 μl of 1 M Tris-hydroxymethylaminomethane, pH 9.5 for neutralization.

(4) Specific binding of IgG from serum/plasma samples to 1.0 ml pre-packed HiTrap™ Protein G columns (Amersham Biosciences, NJ) was according to manufacturer's instructions. Protein G binds to all human IgG subclasses, but does not bind to human IgM. ImmunoPure^® ^(G) IgG Binding Buffer (Pierce) was used as the diluent, binding- and wash buffer. Bound IgG was eluted in 1 ml fractions using ImmunoPure^® ^IgG Elution Buffer (Pierce) (tubes were preloaded with 45 μl of 1 M Tris-hydroxymethylaminomethane, pH 9.5 for neutralization).

### SDS gel electrophoresis

The various Ig fractions were first analysed by ELISA for their MSP-1p42 specific titers, then adjusted to the titer of their source sample (i.e., pre-purification sera/plasma) followed by dilution as follows: all rabbit sera were diluted 1:50 in 1× Tris-Glycine SDS sample buffer (Invitrogen, Carlsbad, CA) and human sera diluted into 1:10 in 1× Tris-Glycine SDS sample buffer for optimal band resolution. Samples were loaded onto 4–20% gradient polyacrylamide Tris-Glycine gels (Invitrogen) and size-fractionated by SDS gel electrophoresis (135 V, 90 min) followed by Coomassie brilliant blue R-250 (Bio-Rad Laboratories, Hercules, CA) staining. In some cases densitometry was performed (Molecular Dynamics Inc., Densitometer Model 375, ImageQuant Fragment Analysis version 1.1a) on the purified bands in order to determine the degree of contamination with other protein bands.

### Determination of protein concentration

The measurements for protein concentration were obtained by absorption at 280 nm using the NanoDrop ND-1000 spectrophotometer (NanoDrop Technologies, Wilmington, DE) and an extinction coefficient of 13.5 (1% IgG solution). The NanoDrop spectrophotometer was chosen for its 1) reduced path length (0.2 mm), making it ideal for measuring small volume samples and 2) high throughput capability.

### ELISA

The titers of antigen-specific antibodies were measured by ELISA in 96-well Immulon 2HB flat bottom microtiter plates (Thermo, Milford, MA). The titers of rabbit sera raised against MSP-1p42 (FVO) were determined using the immunogen, i.e., recombinant MSP-1p42 (FVO). The titers of human plasma obtained from semi-immune Kenyan blood were determined using recombinant MSP-1p42 (3D7). Plates were coated with 0.8 pmol/well of antigen in a volume of 100 μl overnight at 4°C and blocked with 1× PBS with 1% BSA for 1 hr at RT. Serial two-fold dilutions starting at 1:100 for human samples and 1:2,000 for rabbit samples were prepared in 1× PBS/1% BSA and incubated for 2 hrs at 22°C. Bound antibodies were detected for 1 hr at 22°C using alkaline phosphatase-conjugated goat-anti-rabbit (Fc-specific) diluted 1:4,000 or mouse-anti-human (heavy and light chain specific) IgG (Promega, Madison, WI) diluted 1:1,000. Microtiter plates were developed at 22°C for 1 hr using 1 mg/ml of 4-nitrophenyl phosphate disodium salt hexahydrate tablets (PNPP) (Sigma-Aldrich, St. Louis, MO) dissolved in Alkaline Phosphatase Buffer (100 mM Tris-HCl, 100 mM NaCl, 5 mM MgCl_2_) pH 9.5. Titer was defined as the dilution of serum or plasma that yielded an optical density of 1.0 at 405 nm (OD_405 _1.0). In order to correlate data between each assay, a pool of serum from five high-titer Kenyan adults and high-titer sera from MSP-1p42 (FVO) immunized rabbits served as standard plate reference samples.

### Avidity

The avidity of antigen-specific antibodies in serum and purified Ig was measured by indirect competitive ELISA using sodium thiocyanate (NaSCN) as previously described [23,24]. Rabbit sera and human plasma were diluted in 1× PBS/1% BSA according to the OD_405 _1.0 titer value previously determined. The diluted serum was added directly to MSP-1p42-coated wells and incubated for 2 hrs at 22°C. Antigen-antibody interactions were disrupted by the addition of increasing concentrations of the chaotropic agent NaSCN in PBS (ranging from 0.09 to 6 M NaSCN or 1× PBS (reference point) for 30 min at 22°C. Plates were then washed to remove the NaSCN and processed following the standard ELISA protocol. The effective concentration of NaSCN required to release 50% of specific serum antibodies (ED50) was determined and used to compare the avidity of the antibodies recovered by the various purification methods.

### IgG isotype subclass

The immunoglobulin G isotype distribution in human sera [21] and purified Ig was measured by indirect ELISA. Plates were coated overnight at 4°C with 3.2 pmol/well of MSP-1p42 3D7 (final volume, 100 μl/well). Plates were blocked with 300 μl per well of 1× PBS/1% BSA for 1 hr at 37°C. Human sera were diluted 1:40 in 1× PBS/1% BSA and added in serial two-fold dilutions to assay plate. Bound antibodies were detected after 2 hr at 22°C using 1,000-fold diluted alkaline phosphatase-conjugated anti-human (H&L) IgG (Promega) in positive control wells and peroxidase-labeled anti-human IgG1, IgG2, IgG3, and IgG4 (The Binding Site, Birmingham, UK) at dilutions of 1:3,200 for IgG1, IgG2, IgG3 and 1:6,400 for IgG4. Plates were developed by adding 100 μl of a 1:1 ratio of ABTS Peroxidase Substrate Solution A and Peroxidase Substrate Solution B (Kirkegaard and Perry Laboratories, Gaithersburg, MD) and incubating for 1 hr at 22°C. Optical density was measured at 405 nm. Positive isotype responses were reported as any value exceeding the calculated mean OD_405 _value plus two times its standard deviation. For normal human serum the background values for IgG1, IgG2, IgG3, and IgG4 were calculated as 0.36, 0.25, 0.21, and 0.11, respectively.

### Parasite cultures and growth inhibition assays

*Plasmodium falciparum *FVO parasites were maintained asynchronously in media consisting of RPMI 1640 (Invitrogen) with 25 mM HEPES, 7.5% w/v NaHCO_3 _and 10% human pooled serum (blood type A+). Parasites were synchronized using a 40/70% Percoll gradient two days prior to assay setup. Cultures to determine growth inhibition were set up at the schizont stage and continued for one cycle (44 hrs) in 384 well plates. Parasite growth inhibitory activities were measured by quantifying parasite lactate dehydrogenase (pLDH) activities as described previously [25].

## Results

Antibodies exhibit species-specific as well as isotype-specific differences in regards to molecular weight, charge and extent of glycosylation. These differences are expected to affect the quality and efficiency of their recovery when using different purification methods. Non-specific Ig purification methods rely on the chemical properties of Igs to "precipitate" proteins based on their molecular charge or weight (*i.e. *(NH_4_)_2_SO_4_, SEP-EASE), while the "specific" methods rely on the ability of proteins (*i.e*. Protein A, G) to bind the Fc portion of the immunoglobulins.

**Figure 1:**
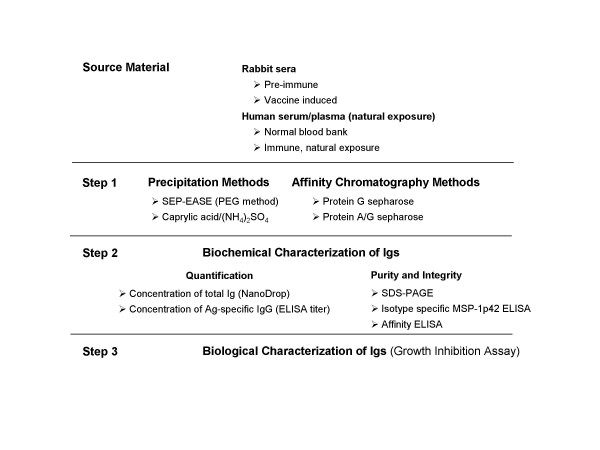
**Procedure overview. **Igs from either rabbit or human serum/plasma were purified (Step 1) and then biochemically characterized in (Step 2), and finally evaluated for the retention of biological activity (Step 3). Each method was tested in at least three independent experiments.

Thus, two precipitation methods and two affinity chromatography-based separation methods were compared for purifying rabbit and human Igs as summarized in the Purification Scheme (Figure 1). Samples used for this comparison were: (1) pooled sera from rabbits immunized with a negative control Ag (reduced and alkylated MSP-1p42 (FVO)); (2) pooled sera from rabbits immunized with MSP-1p42 (FVO); (3) sera from normal blood banked US individuals; and (4) plasma or serum from malaria-experienced donors having high Ab titer against *P. falciparum *MSP-1 induced by natural exposure. First, the purified Igs were quantified for total protein concentration (by 280 nm absorption) and for MSP-1p42-specific antibody-titer (by ELISA). Second, the Ig fractions were analysed for purity and integrity by SDS-gel electrophoresis and for changes in the isotype distribution and the avidity of antibody binding by ELISA. Lastly, the Ig fractions were tested for changes in their biological activity against parasites compared to their respective pre-treatment sample.

### Purity of Ig isolated by the different methods

**Figure 2:**
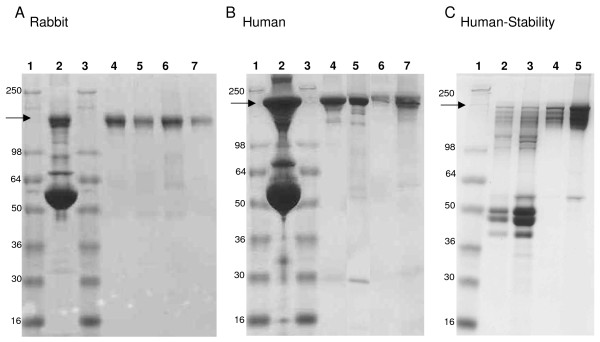
**Ig purity depends on the species of the source material.** MSP-1p42 specific titers of the Ig preparations, Panel A (rabbit samples), Panel B, C (human samples) were determined by ELISA and then adjusted to the pre-purification titer. All rabbit samples were diluted 1:50 and all human samples were diluted 1:10 to achieve optimal band resolution. Lanes: 1) molecular weight marker, 2) source material (pre-purification), 3) MW marker, 4) Protein G-purified Ig, 5) Protein A/G-purified Ig, 6) CA-AS-purified Ig and 7) PEG-purified Igs. Samples in Panel A, B were tested immediately following purification. Samples in Panel C were tested for their stability after storage for 24 weeks at 4°C. Panel C, 1) MW marker, 2) Protein G-purified Ig, 3) Protein A/G-purified Ig, 4) CA-AS-purified Ig and 5) PEG-purified Igs.

Analysis of purified Ig by SDS gel electrophoresis allows the determination of (a) the degree of contamination with other serum proteins and (b) the extent of degradation/proteolytic processing in the recovered Ig fractions (Figure 2). To this end, individual batches of Ig purified by the various purification methods were size-fractionated using SDS-gel electrophoresis followed by analysis of Coomassie Blue staining for the presence of contaminating bands as well as evaluation for purity based on the density of the purified Ig bands. With regard to purity, scanning densitometry revealed that the majority of the protein obtained corresponded to intact Ig, with purity at greater than 95% for all rabbit (Panel A) and human Ig samples (Panel B). Western blots probed with either anti-rabbit Ig alkaline phosphatase conjugate or anti-human Ig-AP conjugate confirmed that all visible lower molecular weight bands reacted with the detecting antibodies thus indicating some proteolysis or degradation of the Ig. For rabbit Igs, all methods yielded high levels of intact pure Igs. For human Igs, Protein G, PEG and CA-AS yielded highly purified intact Ig while the Protein A/G method recovered slightly more proteolysed Igs (Panel B, Lane 5). Based on total yields, the lowest recovery was from the PEG method for the rabbit Ig and from the CA-AS method for the human Ig. Next, the stability of the various Ig preparations was determined since Ig purification is often used to ship and store samples in an attempt to eliminate reported serum/plasma toxicity. For this purpose, human Ig preparations were stored for 24 weeks at 4°C and the SDS gel electrophoresis was repeated (Panel C). While PEG and CA-AS preparation did not show significant degradation, extensive proteolysis was observed in the Protein G and Protein A/G preparations. Table 1 summarizes the total protein concentrations (measured by absorbance at 280 nm); mean ELISA titers, recovery of MSP-1p42 specific IgG (adjusted for volume changes) and the purity of the Ig preparations (determined by densitometry).

**Table 1:** Choice of Purification Method for high yields is dependent on the species.

Sample	Purification method	Average Conc. (mg/mL)^1^	Mean ELISA Titer^2^	Recovery of Ag- specific Igs (%)^3^	Purity of Ig preparation (%)^4^	Stability at 4°C^5^
	Pre-purification		1.3 × 10^6 ^(1.9 × 10^5^)			
Immune Rabbit	CA-AS	12.3	1.4 × 10^6 ^(3.6 × 10^5^)	100	> 95%	NT
	SEP-EASE (PEG)	10.9	1.1 × 10^6 ^(9.6 × 10^4^)	85	> 95%	NT
	Protein A/G	8.9	1.1 × 10^6 ^(6.3 × 10^4^)	84	> 95%	NT
	Protein G	14.3	1.8 × 10^6 ^(2.0 × 10^4^)	100	> 95%	NT

Malaria-Exposed Human	Pre-purification		1.1 × 10^4 ^(962)			
	CA-AS	12.3	1.2 × 10^3 ^(176)	11*	> 95%	Yes
	SEP-EASE (PEG)	14.5	1.6 × 10^4 ^(4 × 10^3^)	100	> 95%	Yes
	Protein A/G	14.4	4.8 × 10^3 ^(95)	44*	>95%	No
	Protein G	19.8	6.6 × 10^3 ^(2.1 × 10^3^)	60	> 95%	No

Data expressed as mean (± SEM) of three independent purification experiments.

^1^Protein concentration was determined by the absorbance of the solution at 280 nm with an extinction coefficient of 13.5 (standard for IgG) for a 1% IgG solution (10 mg/mL)

^2^Mean ELISA titer for an OD_405 _= 1, for rabbits tested against MSP1-p42(FVO) plate antigen, for humans tested against MSP1-p42(3D7) plate antigen

^3^Recovery of Ag-specific Igs is based on Mean ELISA titer.

^4^Determined by scanning densitometry on Coomassie Blue stained gels

^5^Samples were stored for 24 weeks at 4°C and then tested by SDS-gelelectrophoresis, NT = not tested

*A statistically significant difference between the methods in the recovery of Ag-specific Ab by ANOVA. The individual groups were isolated with a Tukey's post test (p < 0.025).

### Efficiency of purification methods differs depending on the species of Ig donors

**Figure 3:**
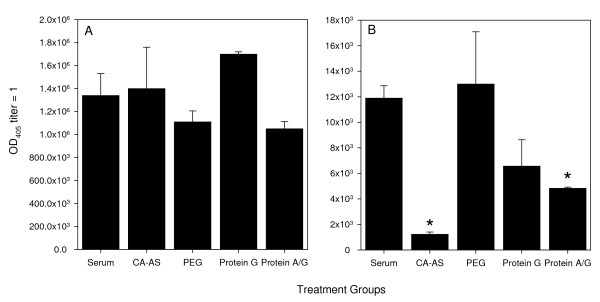
**Changes in Ag-specific titers as a function of purification methods.** Various Ig preparations from (A) rabbit sera or (B) human serum or plasma were tested for changes in the MSP-1p42 specific titer (i.e., OD = 1 at 405 nm). Data shown are the geometric mean and 95% confidence interval of three independent experiments for each purification method. Asterisks indicate statistically significant differences (p < 0.05).

Although the Ig recovery and purity in most cases was high, as shown in Figure 2, the decision was made to characterize the recovered Igs by Ag-specific ELISA assays since protein assays do not distinguish between Igs and any other bands present. This would not only yield information on changes in MSP-1p42 specific IgG titer, but also measure the quality of the recovered Ig, i.e., whether the Igs can still bind to their specific antigen and whether the secondary detecting Ab in the ELISA still recognizes the IgG (Figure 3). Comparison of Ag-specific IgG titers in the various Ig preparations as a function of the purification method did not distinguish significant differences when using rabbit sera as source material, albeit PEG and Protein A/G consistently trended toward lower MSP-1p42-specific titers. However, for human IgG preparations purified by the different methods, significantly lower titers in Igs purified with either CA-AS or Protein A/G Sepharose were observed (p = 0.025, One way ANOVA followed by Tukey post-test). PEG reliably yielded the highest recovery of MSP-1p42 specific human Ab.

### Impact of purification methods on the isotype subclass distribution in Ig preparations

**Figure 4:**
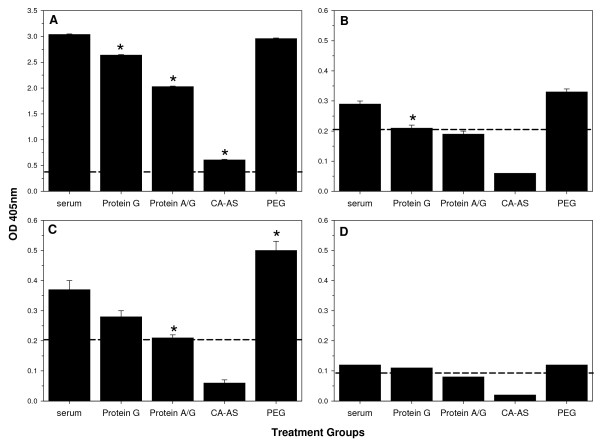
**Effect of purification methods on isotype subclass distribution in Ig preparations.** Ig preparations purified using the various methods were tested for changes in MSP-1p42 specific IgG1 (Panel A), IgG2 (Panel B), IgG3 (Panel C) and IgG4 (Panel D) for each treatment; pre-treatment serum, Protein G, Protein A/G, CA-AS, and PEG. Immune serum was from a naturally exposed individual residing in Gabon. Data shown are the mean OD405 (± SEM) of three independent isotype subclass specific ELISA experiments. Asterisks indicate statistically significant differences (p < 0.05). Dashed lines indicate background level responses for each isotype subclass test.

Immunoglobulins have distinct biological functions which are governed by the heavy chain of the Ab molecule. The heavy chain differs between Ig isotypes and mediates binding to isotype-specific Fc receptors and/or binding and activation of complement. Thus, a method used for purifying functional antibodies should not bias the purified Ab isotype profile. To this end, the four selected purification methods were evaluated for causing a change in the isotype profile (Figure 4). Ig preparations obtained after purifying malaria-immune sera were analysed in a MSP-1p42 specific ELISA followed by an isotype subclass specific detection step using secondary antibodies against IgG1, IgG2, IgG3 or IgG4. While Protein G and PEG did not show any relative bias in the separation of MSP-1p42 specific IgG antibodies, not all methods were equivalent relative to their ability to recover all isotype subclasses. Only with the PEG method no significant change for IgG1, IgG2, and IgG4 was detected, while for IgG3, a significantly higher proportion of MSP-1p42 specific Ab was detected compared to the pre-treated source material (Figure 4).

### Purification methods do not affect the avidity of MSP-1p42 specific Ab

CA-AS as well as affinity chromatography using Protein A/G or G alone involved adjustment of the sample to a low pH at some point during the purification process, which may result in the degradation of the Ig or affect Ag-recognition. Therefore, the purified human and rabbit Igs were tested for their binding strength to their specific Ag (MSP-1p42) using an "avidity ELISA" which employed the chaotropic agent NaSCN. Samples from three independent purification experiments were tested and no changes in the binding avidity (ED_50 _= 2.0 M for both, rabbit and human sample) were observed, regardless of the purification method.

### Purification methods can affect the biological activity of MSP-1p42 specific Ig

**Figure 5:**
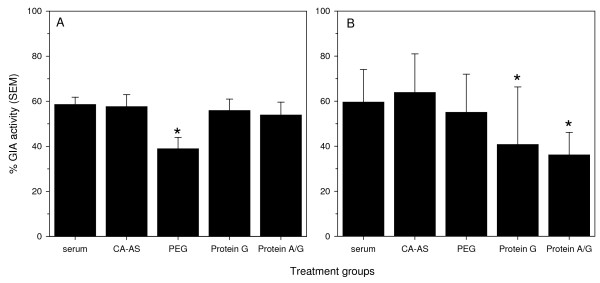
**Growth inhibitory activity of various Ig preparations from (A) MSP-1p42 (FVO) specific rabbit sera or (B) human malaria-experienced plasma was measured against FVO parasites in a pLDH based growth inhibition assay (GIA).** Data are expressed as the mean and SEM of three independent purifications per method (sample size for all treatment groups n = 27 except for Protein A/G (n = 6)). Asterisk indicates statistically significant differences (p < 0.01).

Finally, it was determined whether any of the selected purification methods altered the biological function of the isolated Ig (Figure 5). The concentration of MSP-1p42 specific Ig from each process was adjusted to the same titer as its pre-treatment serum/plasma sample and then tested for retention of its *in vitro *parasite growth inhibitory activity using a pLDH based GIA assay [25]. Differences in the concentration of Ag-specific Ig due to the efficiency of the purification method were excluded by normalizing the samples based on their ELISA Ab titers. As a result, only changes in the biological activity as a function of the method would be revealed. When comparing the activities in rabbit Ig preparations, the only method that negatively impacted GIA activity was PEG (p = 0.006 Kruskal Wallis with Dunn's post test for the differences between the methods and only PEG yielded lower biological activity compared to the source material p < 0.001). For human Ig preparations (Figure 5, Panel B), Protein G and Protein A/G were inferior to the other methods tested (differences between the methods p < 0.001 ANOVA with Tukey's post-test, Protein G and Protein A/G identified as different from source material by paired T-test, p < 0.001).

## Discussion

Many disease models lack correlates of immunity and protection against infection. In models where a role for antibodies has been established, efforts are focused toward characterizing protective humoral immune responses. The need to enrich or purify Ig rises from the fact that contaminants in serum or plasma often interfere with *in vitro *readout methods that are used to characterize immune responses. Examples of contaminants that can influence functional biological activities, either negatively or positively are blood-borne pathogens, oxidized lipids, chemokines, therapeutic drugs, herbal remedies as well as other serum factors. Thus, elimination of these potential effectors is essential in order to identify target protective Igs.

The ability to purify Ig from sera or plasma from immune animals or humans with a high yield and without selective loss of isotypes is imperative when conducting analytical experiments. Apart from the *in vitro *analysis of humoral immune responses, Igs are frequently batch-purified from serum for the purpose of passive transfer of the Ig as therapeutic agents into naive animals and even human hosts. In either case, it is imperative that the purification method does not introduce artifacts, such as a bias in the Ig isotype, or alter the functional activity of the antibody molecules.

In the present study, four frequently used Ig purification methods were evaluated on the basis of purity of the product, antibody specificity, isotype profile and functionality using rabbit sera and human serum/plasma as the source material. The results showed that the choice of the optimal purification method depended upon the species from which the sera/plasma was derived. When taking into consideration both yield and purity, we concluded that the highest quality Ig could be achieved using either Protein G, Protein A/G, or CA-AS and that these same methods were also the optimal Ig purification methods relative to high-level Ag-specific Ig recovery and functionality for rabbit IgG. For human Ig, PEG-Ig partitioning under optimal conditions lead to concentrated Igs, having overall higher recovery rates, enhanced specific activities and yields greater than 100% presumably due to separation from inhibitors present in the serum/plasma. This was specifically shown by the preferential enrichment of the IgG3 as well as the 100% recovery of IgG1, IgG2 and IgG4 subclass molecules. Both Protein G and Protein A/G purification methods yielded statistically less functional antibody activities compared to the source material. This may have been caused by the, albeit transient, harsh elution conditions which – in some cases – have been shown to reduce antibody titer, decrease immunoreactivity and distort the antibody structure. Concomitantly, the retention of functional antibody activities by the PEG method may be due to the nondenaturing and reversible interactions of the water soluble, non-ionic PEG polymers with Igs [26]. No change in Ab avidity for human and rabbit Ig by any of the purification methods used in this study were observed. While human Igs enriched by PEG and CA-AS were stable when stored for extended time at 4°C, Igs purified by Protein A/G or Protein G degraded (Figure 2C). Finally, although not specifically evaluated here, since the role for IgM isotype in inhibitory activities against parasites has not been defined as yet, only the methods that preserve and retain these Ig specificities will be useful to meeting this goal.

## Conclusion

In conclusion, some considerable qualitative and quantitative differences between the methods were detected that were evaluated and, thus, Ig purification methods must be carefully selected based on not only the animal species from which the serum or plasma was derived, but with regard to its intended use. For most purposes, the action of specific antibody isotypes may not be well known thus preservation of isotype profiles remains critical, herein, to establishing antibody immune correlates of protection.

## Authors' contributions

ESBL drafted the manuscript, co-designed the study, conducted some of the purifications, performed growth inhibition assays, analyzed the data and did the statistical evaluation. RMM conducted some of the purifications and developed the isotype-specific ELISA and performed all of the ELISA experiments. EHD conducted some of the purifications, assisted in growth inhibition assays and analysed the samples by gel electrophoresis. FK worked on the quantification of purified immunoglobulins and did some of the preliminary work on the PEG method. JW provided some of the human samples. EA designed the study, directed the work and edited the manuscript. All authors have read and approved the final manuscript.

## Disclaimer

Research was conducted in compliance with the Animal Welfare Act and other federal statutes and regulations relating to animals and experiments involving animals and adheres to principles stated in the *Guide for the Care and Use of Laboratory Animals*, NRC Publication, 1996 edition. Human blood for plasma from volunteers in western Kenya was obtained under Approved Protocol # 954.

The authors' views are private and are not to be construed as official policy of the Department of Defense or the U.S. Army. This work was supported by the United States Agency for International Development, Project Number 936-6001, Award Number AAG-P-00-98-00006, Award Number AAG-P-00-98-00005, and by the United States Army Medical Research and Materiel Command.

## References

[B1] Buchacher A, Iberer G (2006). Purification of intravenous immunoglobulin G from human plasma – aspects of yield and virus safety. Biotechnol J.

[B2] Chang CE, Eo HG, Lee YS, Chung SK, Shin JS, Lah YK, Park CW, Jung JT, Huh JW, Lee SM (2000). Human intravenous immunoglobulin preparation and virus inactivation by pasteurization and solvent detergent treatment. Prep Biochem Biotechnol.

[B3] Singh S, Miura K, Zhou H, Muratova O, Keegan B, Miles A, Martin LB, Saul AJ, Miller LH, Long CA (2006). Immunity to recombinant *Plasmodium falciparum *merozoite surface protein 1 (MSP1): protection in *Aotus nancymai *monkeys strongly correlates with anti-MSP1 antibody titers and *in vitro *parasite-inhibitory activity. Infect Immun.

[B4] Parkkinen J, Rahola A, von Bonsdorff L, Tolo H, Torma E (2006). A modified caprylic acid method for manufacturing immunoglobulin G from human plasma with high yield and efficient virus clearance. Vox Sang.

[B5] Turpin EA, Lauer DC, Swayne DE (2003). Development and evaluation of a blocking enzyme-linked immunosorbent assay for detection of avian metapneumovirus type C-specific antibodies in multiple domestic avian species. J Clin Microbiol.

[B6] Temponi M, Kageshita T, Perosa F, Ono R, Okada H, Ferrone S (1989). Purification of murine IgG monoclonal antibodies by precipitation with caprylic acid: comparison with other methods of purification. Hybridoma.

[B7] Eliasson M, Olsson A, Palmcrantz E, Wiberg K, Inganäs M, Guss B, Lindberg M, Uhlén M (1988). Chimeric IgG-binding receptors engineered from staphylococcal protein A and streptococcal protein G. J Biol Chem.

[B8] Bjorck L, Kronvall G (1984). Purification and some properties of streptococcal protein G, a novel IgG-binding reagent. J Immunol.

[B9] Russ C, Callegaro I, Lanza B, Ferrone S (1983). Purification of IgG monoclonal antibody by caprylic acid precipitation. J Immunol Meth.

[B10] Roque ACA, Silva CSO, Taipa MA (2007). Affinity-based methodologies and ligands for antibody purification: Advances and perspectives. J Chromatogr A.

[B11] Azevedo AM, Rosa PA, Ferreira IF, Aires-Barros MR (2007). Optimisation of aqueous two-phase extraction of human antibodies. J Biotechnol.

[B12] Wilson S, Booth M, Jones FM, Mwatha JK, Kimani G, Kariuki HC, Vennervald BJ, Ouma JH, Muchiri E, Dunne DW (2007). Age-adjusted *Plasmodium falciparum *antibody levels in school-aged children are a stable marker of microgeographical variations in exposure to Plasmodium infection. BMC Infect Dis.

[B13] Verhofstede C, Van Gelder P, Rabaey M (1988). The infection-stage-related IgG response to *Toxoplasma gondii *studied by immunoblotting. Parasitol Res.

[B14] Morgan J, Dias JC, Gontijo ED, Bahia-Oliveira L, Correa-Oliveira R, Colley DG, Powell MR (1996). Anti-*Trypanosoma cruzi *antibody isotype profiles in patients with different clinical manifestations of Chagas' disease. Am J Trop Med Hyg.

[B15] Soe S, Theisen M, Rouissilhon C, Aye KS, Druilhe P (2004). Association between protection against clinical malaria and antibodies to merozoite surface antigens in an area of hyperendemicity in Myanmar: complementarity between responses to merozoite surface protein 3 and the 220-kilodalton glutamate-rich protein. Infect Immun.

[B16] Rouissilhon C, Ouevray C, Mueller-Graf C, Tall A, Rogier C, Trape JF, Theisen M, Balde A, Perignon JL, Druilhe P (2007). Long-term clinical protection from falciparum malaria is strongly associated with IgG3 antibodies to merozoite surface protein 3. PLoS Med.

[B17] Jafarshad A, Dziegiel MH, Lundquist R, Nielsen LK, Singh S, Druilhe P (2007). A novel antibody-dependent cellular cytotoxicity mechanism involved in defense against malaria requires costimulation of monocytes FcgammaRII and FcgammaRIII. J Immunol.

[B18] Tebo AE, Kremsner PG, Luty AJ (2001). *Plasmodium falciparum*: a major role for IgG3 in antibody-dependent monocyte-mediated cellular inhibition of parasite growth *in vitro*. Exp Parasitol.

[B19] Frank MB, Frank MB (2001). Antibody binding to Protein A and Protein G beads. Molecular Biology Protocols, Oklahoma City.

[B20] Darko CA, Angov E, Collins WE, Bergmann-Leitner ES, Girouard AS, Hitt SL, McBride JS, Diggs CL, Holder AA, Long CA, Barnwell JW, Lyon JA (2005). The clinical-grade 42-kilodalton fragment of merozoite surface protein 1 of *Plasmodium falciparum *strain FVO expressed in Escherichia coli protects Aotus nancymai against challenge with homologous erythrocytic-stage parasites. Infect Immun.

[B21] Lyon JA, Carter JM, Thomas AW, Chulay JD (1997). Merozoite surface protein-1 epitopes recognized by antibodies that inhibit *Plasmodium falciparum *merozoite dispersal. Mol Biochem Parasitol.

[B22] Perosa F, Carbone R, Ferrone S, Dammacco F (1990). Purification of human immunoglobulins by sequential precipitation with caprylic acid and ammonium sulphate. J Immunol Methods.

[B23] Pullen GR, Fitzgerald MG, Hosking CS (1986). Antibody avidity determination by ELISA using thiocyanate elution. J Immunol Methods.

[B24] Bergmann-Leitner ES, Scheiblhofer S, Weiss R, Duncan EH, Leitner WW, Chen D, Angov E, Khan F, Williams JL, Winter DB, Thalhamer J, Lyon JA, Tsokos GC (2005). C3d binding to the circumsporozoite protein carboxy-terminus deviates immunity against malaria. Int Immunol.

[B25] Bergmann-Leitner ES, Duncan EH, Burge JR, Spring M, Angov E (2008). Short report: Miniaturization of a high-hroughput pLDH-based *Plasmodium falciparum *growth inhibition assay for small volume samples from preclinical and clinical vaccine trials. Am J Trop Med Hyg.

[B26] Ingham KC (1984). Protein precipitation with polyethylene glycol. Methods Enzymol.

